# Apathy/depression, but not subjective fatigue, is related with cognitive dysfunction in patients with multiple sclerosis

**DOI:** 10.1186/1471-2377-14-3

**Published:** 2014-01-06

**Authors:** Masaaki Niino, Nobuhiro Mifune, Tatsuo Kohriyama, Masahiro Mori, Takashi Ohashi, Izumi Kawachi, Yuko Shimizu, Hikoaki Fukaura, Ichiro Nakashima, Susumu Kusunoki, Katsuichi Miyamoto, Kazuto Yoshida, Takashi Kanda, Kyoichi Nomura, Takashi Yamamura, Fumihito Yoshii, Jun-ichi Kira, Shunya Nakane, Kazumasa Yokoyama, Makoto Matsui, Yusei Miyazaki, Seiji Kikuchi

**Affiliations:** 1 Department of Clinical Research, Hokkaido Medical Center, Yamanote 5jo 7chome, Nishi-ku, Sapporo 063-0005, Japan; 2 School of Management, Kochi University of Technology, Kochi, Japan; 3 Department of Neurology, Hiroshima City Hospital, Hiroshima, Japan; 4 Department of Neurology, Graduate School of Medicine, Chiba University, Chiba, Japan; 5 Department of Neurology, Tokyo Women’s Medical University Yachiyo Medical Center, Chiba, Japan; 6 Department of Neurology, Brain Research Institute, Niigata University, Niigata, Japan; 7 Department of Neurology, Tokyo Women’s Medical University School of Medicine, Tokyo, Japan; 8 Department of Neurology, Iwate Medical School, Morioka, Japan; 9 Department of Neurology, Saitama Medical Center, Saitama Medical University, Saitama, Japan; 10 Department of Neurology, Tohoku University School of Medicine, Sendai, Japan; 11 Department of Neurology, Kinki University School of Medicine, Osaka, Japan; 12 Department of Neurology, Asahikawa Red Cross Hospital, Asahikawa, Japan; 13 Department of Neurology and Clinical Neuroscience, Yamaguchi University Graduate School of Medicine, Yamaguchi, Japan; 14 Department of Immunology, National Institute of Neuroscience, National Center of Neurology and Psychiatry, Tokyo, Japan; 15 Department of Neurology, Tokai University School of Medicine, Kanagawa, Japan; 16 Department of Neurology, Neurological Institute, Graduate School of Medical Sciences, Kyushu University, Fukuoka, Japan; 17 Department of Clinical Research, Nagasaki Kawatana Medical Center, Nagasaki, Japan; 18 Department of Neurology, Juntendo University School of Medicine, Tokyo, Japan; 19 Department of Neurology, Kanazawa Medical University, Ishikawa, Japan; 20 Department of Neurology, Hokkaido Medical Center, Sapporo, Japan

**Keywords:** Multiple sclerosis, Cognition, Apathy, Fatigue, Depression, Japanese

## Abstract

**Background:**

Cognitive impairment could affect quality of life for patients with multiple sclerosis (MS), and cognitive function may be correlated with several factors such as depression and fatigue. This study aimed to evaluate cognitive function in Japanese patients with MS and the association between cognitive function and apathy, fatigue, and depression.

**Methods:**

The Brief Repeatable Battery of Neuropsychological tests (BRB-N) was performed in 184 Japanese patients with MS and 163 healthy controls matched for age, gender, and education. The Apathy Scale (AS), Fatigue Questionnaire (FQ), and Beck Depression Inventory Second Edition (BDI-II) were used to evaluate apathy, fatigue, and depression, respectively. Student’s t-test was used to compare MS patients and healthy controls. Correlations between two factors were assessed using the Pearson correlation test, and multiple regression analysis was used to evaluate how much each factor affected the BRB-N score.

**Results:**

In all BRB-N tests, patients with MS scored significantly lower than controls, and the effect size of symbol digit modalities test was the highest among the 9 tests of the BRB-N. Patients with MS had higher AS (*p* < 0.001), FQ (*p* < 0.0001), and BDI-II (*p* < 0.0001) scores than controls. In patients with MS, scores on most of the BRB-N tests correlated with scores on the AS and BDI-II; however, there was little correlation between scores on the BRB-N tests and those on the FQ.

**Conclusions:**

Cognitive function was impaired, particularly information-processing speed, and decreased cognitive function was correlated with apathy and depression in Japanese patients with MS. Despite the association between cognitive variables and depression/apathy, cognitive function was impaired beyond the effect of depression and apathy. However, subjective fatigue is not related with cognitive impairment. Taken together, this suggests that different therapeutic approaches are needed to improve subjective fatigue and cognition, and thereby quality of life, in patients with MS.

## Background

The prevalence of cognitive dysfunction in multiple sclerosis (MS) has been historically underestimated due to difficulty in detecting cognitive impairment during brief office visits without performing a formal neuropsychological assessment and a widespread belief that cognitive dysfunction occurs rarely and then only in the advanced stages of the disease [1]. However, in neuropsychological studies, 40–65% of MS patients show cognitive impairment with prominent involvement of memory, sustained attention, and information processing speed [2]. Prevalence of cognitive dysfunction in MS varies among studies depending on the type of tests used and whether the studies are based in community or clinical settings, with clinical settings showing higher rates [3]. To evaluate cognitive deficits in MS, a focused measure of cognitive abilities using the Brief Repeatable Battery of Neuropsychological tests (BRB-N) was developed [4,5]. The BRB-N was originally written in English, and has been translated into other languages including Dutch [6] and Spanish [7]. Test scores on the BRB-N are influenced by variables such as age, gender, and level of education [6,8], and the BRB-N was shown to have a sensitivity of 71% and a specificity of 94% in discriminating MS patients with and without cognitive impairment [9].

Apathy has been defined as lack of motivation not attributable to diminished level of consciousness, cognitive impairment, or emotional distress, and the three domains of apathy are considered to be “deficits in goal-directed behavior”, “a decrement in goal-related thought content”, and “emotional indifference with flat affect” [10]. Fatigue is a frequent complication of MS, and MS patients often report that fatigue impairs their cognitive function. However, the relation between fatigue and cognitive performance is complex and inconsistent [11]. Depression is also a common symptom of MS, and recent studies suggest that information processing speed, working memory, and executive functioning of cognitive function may indeed be affected in patients with moderate to severe depression [12].

It is important for patient management to detect cognitive impairment accurately. Further, the relationship between cognitive impairment and the emergence of neuropsychiatric disorders in patients with MS remains unclear, and apathy, fatigue, and depression have not been investigated in Japanese patients with MS. The aim of this study was to evaluate cognitive function in Japanese patients with MS, and the association between cognitive function and fatigue, apathy, and depression.

## Methods

### Patients with MS and healthy individuals

This study was conducted between November 2010 and March 2012 with 184 Japanese patients with MS (female/male = 135/49) diagnosed using the 2005 revised McDonald criteria [13] at 18 sites (Hiroshima City Hospital, Chiba University, Tokyo Women’s Medical University Yachiyo Medical Center, Niigata University, Tokyo Women’s Medical University School of Medicine, Saitama Medical School, Tohoku University Graduate School of Medicine, Kinki University School of Medicine, Asahikawa Red Cross Hospital, Yamaguchi University Graduate School of Medicine, National Center of Neurology and Psychiatry, Tokai University School of Medicine, Kyushu University, Nagasaki Kawatana Medical Center, Iwate Medical University, Juntendo University School of Medicine, Kanazawa Medical University, and Hokkaido Medical Center) in Japan. Patients with neuromyelitis optica (NMO) or NMO spectrum disorders were excluded from this study. Patients were categorized according to MS subtype: 2 had primary progressive, 167 had relapsing–remitting, and 15 had secondary progressive disease, and did not experience relapses for at least 1 month before participating in this study. The mean age of the MS patients was 39.3 years (SD. 10.1; range 18–71 years). Duration of compulsory education in Japan is 9 years and the mean duration of education excluding compulsory education in this sample was 4.92 years (SD. 1.83; range 0–9 years). The mean age at onset was 30.0 years (SD. 10.1) and the mean duration of disease was 9.3 years (SD. 7.2). The mean Expanded Disability Status Scale (EDSS) was 2.38 (SD. 2.04; range 0–8.5). Among 184 patients with MS, 109 patients received interferon β (IFNβ) as disease modifying drugs (DMDs) when they participated in this study. Twenty-five patients received other DMDs such as fingolimod and natalizumab, and 50 patients did not receive any DMDs. A total of 163 healthy controls (female/male = 119/44) participated in this study. The mean age of the healthy controls was 39.2 years (SD. 11.9; range 19–76 years). The mean duration of education excluding compulsory education was 5.15 years (SD. 2.08; range 0–13 years). Differences in sex ratio, duration of education, and age at examination between the patients and controls were not significant (*p* > 0.05). People with diseases of the central nervous system or major medical illnesses were excluded from the healthy control group. All participants had adequate vision to complete testing. The study protocol was approved by the ethics committee of each participating site, and all patients and healthy controls gave their written informed consent to participate in the study.

### Battery for neuropsychological evaluation, apathy, fatigue, and depressive state

#### Assessment of cognitive function

For neuropsychological evaluation, patients and healthy individuals completed the BRB-N, which includes tests of verbal learning and memory (selective reminding test, SRT), visuospatial memory and learning (10/36 spatial recall test, SPART), attention, information processing, and working memory (paced auditory serial addition test, PASAT, and symbol digit modalities test, SDMT), and verbal fluency (word list generation test, WLG). The BRB-N, which was originally written in English, was translated into Japanese and used for assessment of neuropsychological functions. The test battery was administered in the following order: SRT, SPART, SDMT, PASAT, delayed recall of the SRT (SRT-D), delayed recall of the SPART (SPART-D), and WLG. Scores derived from these tests included long-term storage (SRT-LTS), consistent long-term retrieval (SRT-CLTR), and delayed recall (SRT-D) from the SRT, immediate recall (SPART) and delayed recall (SPART-D) from the SPART, total score from the SDMT, PASAT 2-second and 3-second versions (PASAT2 and PASAT3), and total score from the WLG test.

#### Assessment of apathy

Apathy was measured using the Apathy Scale (AS), which is an abridged version of an apathy scale designed by Robert Marin [14], with some modifications [15]. Briefly, patients were provided with four possible answers to 14 questions: “not at all”, “slightly”, “some”, and “a lot”. Each score ranged from 0 to 42 and higher scores indicated more severe apathy [15]. The AS was translated into Japanese and had been used previously in a study of Japanese patients with stroke [16].

#### Assessment of fatigue

In 1989, Krupp et al. reported data of fatigue in MS using the Fatigue Severity Scale [17]. The group expanded the scale of the Fatigue Questionnaire (FQ) and administered the FQ to a large group of medical and psychiatric patients [18]. The FQ, which was translated into Japanese and has been used previously [19], was used to measure fatigue in patients with MS. The FQ consists of 29 items each of which is a statement about fatigue and is rated from 1 representing “completely disagree” to 7 representing “completely agree”, with a higher score indicating more fatigue [18]. Mean scores were calculated for each patient.

#### Assessment of depression

The Beck Depression Inventory second edition (BDI-II), which consists of 21 items rated on a scale from 0 to 3, is a valid and reliable measure of depressive state [20]. The Japanese version of the BDI-II, which was developed to be able to assess depressive symptoms in Japanese people, is psychometrically robust [21], and was used for evaluation of depression in the present study.

### Statistical analysis

Statistical analyses were performed using the SAS 9.3 software package (SAS Institute Inc., Cary, NC). For analysis, raw data for the 9 tests (SRT-LTS, SRT-CLTR, SRT-D, SPART, SPART-D, SDMT, PASAT3, PASAT2, and WLG) were used, and scores for each of these tests were standardized as a mean score of 0 and standard deviation of 1. Student’s t-test was used to compare average data between MS patients and healthy controls or between MS patients who received IFNβ and those who did not receive IFNβ. Correlations between two factors were assessed using the Pearson correlation test. Multiple regression analysis was used to evaluate how much each factor—patient, AS score, FQ score, and BDI-II score—affected the BRB-N score. *p* values less than 0.05 were considered statistically significant.

## Results

### BRB-N data in MS patients and healthy controls

Cronbach's alpha coefficients for all 9 BRB-N test scores were 0.93 in MS patients and 0.82 in the healthy control group, suggesting a high level of confidence. Thus, the BRB-N translated into Japanese showed a high internal consistency for each category and all scores. Table 1 shows mean BRB-N scores in MS patients and healthy controls. Table 2 shows a significant negative correlation between age at examination and each of the BRB-N components, except for WLG, was found in healthy controls. Negative correlations between duration of education and SRT-LTS, SRT-CTLR, SRT-D, SDMT, and PASAT2 were found in healthy controls, although there were no correlations between score and duration of education in the other 4 tests. In all 9 tests, scores were significantly lower in MS patients than in healthy controls. Table 2 also shows the standardized scores for each test in patients and healthy controls. To evaluate which test score is most different between patients and healthy controls, effect size (Cohen’s *d*) was calculated. It was found that SDMT had the greatest effect size (1.34) of the 9 items (SRT-LTS, 0.67; SRT-CLTR, 0.72; SRT-D, 0.67; SPART, 0.86; SPART-D, 0.67; PASAT3, 0.95; PASAT2, 0.96; and WLG, 0.95). In the comparison of MS patients who received IFNβ and those who did not receive IFNβ, there were not any significant differences in all 9 BRB-N tests between the two groups.

**Table 1:** Mean BRB-N scores in patients with MS and healthy controls

**BRB-N**	**MS patients**		**Healthy controls**	
	**Raw scores**	**Standardized scores**	**Raw scores**	**Standardized scores**
SRT-LTS	40.85 ± 17.18 (0–72)	−0.30 ± 1.10	50.75 ± 11.68 (14–70)	0.34 ± 0.75
SRT-CLTR	31.43 ± 18.68 (0–72)	−0.32 ± 1.04	43.60 ± 14.58 (2–70)	0.36 ± 0.81
SRT-D	7.99 ± 3.07 (0–12)	−0.30 ± 1.13	9.71 ± 1.87 (5–12)	0.34 ± 0.69
SPART	18.91 ± 5.51 (5–30)	−0.37 ± 1.00	23.26 ± 4.55 (10–30)	0.42 ± 0.83
SPART-D	6.85 ± 2.34 (0–10)	−0.30 ± 1.05	8.26 ± 1.85 (1–12)	0.34 ± 0.83
SDMT	46.20 ± 15.30 (4–84)	−0.52 ± 0.94	64.30 ± 11.24 (37–91)	0.59 ± 0.69
PASAT3	40.83 ± 15.44 (0–60)	−0.40 ± 1.14	52.45 ± 7.26 (24–60)	0.45 ± 0.54
PASAT2	30.18 ± 14.02 (0–60)	−0.41 ± 1.06	41.55 ± 8.94 (18–60)	0.46 ± 0.68
WLG	21.95 ± 7.21 (2–37)	−0.40 ± 1.02	27.99 ± 5.29 (12–40)	0.45 ± 0.75

For each test, data are expressed as mean ± standard deviation scores (ranges). MS patient scores were significantly different from healthy control scores for all tests (*p* < 0.0001).

**Table 2:** Correlation between age at examination or duration of education and the BRB-N

	**Age at examination**				**Duration of education**			
	**MS patients**		**Healthy controls**		**MS patients**		**Healthy controls**	
**BRB-N**	**γ**	***p *** **value**	**γ**	***p *** **value**	**γ**	***p *** **value**	**γ**	***p *** **value**
SRT-LTS	−0.23	0.0017	−0.53	<0.0001	0.21	0.0045	0.22	0.0055
SRT-CLTR	−0.25	0.0006	−0.55	<0.0001	0.17	0.0214	0.19	0.0155
SRT-D	−0.16	0.0318	−0.53	<0.0001	0.17	0.0199	0.21	0.0084
SPART	−0.22	0.0023	−0.32	<0.0001	0.11	n.s.	0.07	n.s.
SPART-D	−0.24	0.0009	−0.25	0.0011	0.07	n.s.	0.06	n.s.
SDMT	−0.24	0.0012	−0.44	<0.0001	0.12	n.s.	0.23	0.0027
PASAT3	−0.13	n.s.	−0.25	0.0014	0.13	n.s.	0.15	n.s.
PASAT2	−0.10	n.s.	−0.31	<0.0001	0.12	n.s.	0.19	0.0150
WLG	−0.10	n.s.	−0.11	n.s.	0.04	n.s.	−0.09	n.s.

n.s.: not significant (*p* > 0.05).

### Correlation of disease duration or EDSS with BRB-N in MS patients

Table 3 shows that in each of the 9 tests except the WLG, a significant but weak negative correlation was found between disease duration and score. On the other hand, relatively strong negative correlations were found between the EDSS and BRB-N scores in MS patients.

**Table 3:** Correlation between disease duration or EDSS score and BRB-N test scores in patients with MS

	**Disease duration**		**EDSS**	
**BRB-N**	**γ**	***p *** **value**	**γ**	***p *** **value**
SRT-LTS	−0.16	0.0271	−0.37	<0.0001
SRT-CLTR	−0.19	0.0093	−0.34	<0.0001
SRT-D	−0.18	0.0120	−0.37	<0.0001
SPART	−0.22	0.0023	−0.25	0.0005
SPART-D	−0.24	0.0010	−0.28	0.0002
SDMT	−0.18	0.0133	−0.49	<0.0001
PASAT3	−0.24	0.0012	−0.42	<0.0001
PASAT2	−0.18	0.0141	−0.40	<0.0001
WLG	−0.09	n.s.	−0.33	<0.0001

n.s.: not significant (*p* > 0.05).

### Apathy, fatigue, and depression in MS patients and healthy controls

Mean scores on the AS, FQ, and BDI-II in MS patients were 14.38 ± 6.98 (range, 0–34), 3.89 ± 1.18 (range, 1.00–7.24), and 13.54 ± 9.32 (range, 0–45), respectively. Corresponding scores for healthy controls were 12.03 ± 5.55 (range, 0–27), 3.40 ± 0.89 (range, 1.00–5.41), and 9.47 ± 6.59 (range, 0–27). For all 3 instruments, MS patients scored significantly higher compared to healthy controls (*p* = 0.0007, *p* < 0.0001, and *p* < 0.0001, respectively), suggesting the presence of more apathy, more fatigue, and more depression in patients. In MS patients, AS, FQ, and BDI-II scores were not associated with disease duration. On the other hand, positive correlations were noted between scores on the AS, FQ, or BDI-II and the EDSS in MS patients (γ = 0.17, *p* < 0.05; γ = 0.15, *p* < 0.05; and γ = 0.20, *p* < 0.01; respectively).

### Relationship between cognitive performance and measures of apathy, fatigue, and depression

Next we evaluated whether apathy, fatigue, and depression were correlated with the BRB-N. Table 4 shows that in healthy controls, AS and FQ scores were not correlated with BRB-N scores. However, SRT-LTS, SRT-CLTR, SDMT, PASAT3, and PASAT2 scores were correlated with BDI-II score. On the other hand, in patients with MS, most test scores of the BRB-N were correlated with the scores on the AS and BDI-II. However, FQ score was not correlated with any of the BRB-N tests except WLG.

**Table 4:** Correlation between apathy (apathy scale), fatigue (fatigue questionnaire), and depression (BDI-II) and the BRB-N

	**Apathy**				**Fatigue**				**Depression**			
	**MS patients**		**Healthy controls**		**MS patients**		**Healthy controls**		**MS patients**		**Healthy controls**	
**BRB-N**	**γ**	***p *** **value**	**γ**	***p *** **value**	**γ**	***p *** **value**	**γ**	***p *** **value**	**γ**	***p *** **value**	**γ**	***p *** **value**
SRT-LTS	−0.23	0.0018	−0.04	n.s.	0.05	n.s.	0.02	n.s.	−0.18	0.0208	−0.18	0.0226
SRT-CLTR	−0.22	0.0031	−0.04	n.s.	0.04	n.s.	0.02	n.s.	−0.13	n.s.	−0.16	0.0370
SRT-D	−0.23	0.0014	0.00	n.s.	0.05	n.s.	0.01	n.s.	−0.14	n.s.	−0.11	n.s.
SPART	−0.27	0.0003	−0.00	n.s.	−0.02	n.s.	−0.09	n.s.	−0.18	0.0185	−0.02	n.s.
SPART-D	−0.33	<0.0001	−0.03	n.s.	−0.01	n.s.	−0.04	n.s.	−0.16	0.0446	−0.08	n.s.
SDMT	−0.28	0.0002	−0.07	n.s.	−0.03	n.s.	−0.01	n.s.	−0.28	0.0002	−0.29	0.0002
PASAT3	−0.22	0.0033	0.12	n.s.	−0.04	n.s.	−0.06	n.s.	−0.25	0.0013	−0.21	0.0083
PASAT2	−0.21	0.0047	0.01	n.s.	−0.04	n.s.	−0.14	n.s.	−0.23	0.0031	−0.29	0.0002
WLG	−0.23	0.0016	−0.07	n.s.	0.16	0.03	0.03	n.s.	−0.15	0.0458	−0.15	n.s.

n.s.: not significant (*p* > 0.05).

### Effect of patient, apathy, fatigue, and depression in the BRB-N

To examine how much each of the patient, apathy, fatigue, and depression factors affect the BRB-N score, multiple regression analysis was conducted with these 4 factors as explanatory variables for each BRB-N test. In this analysis, “patient” was defined as 1 and “healthy control” as 0. It was found that only “patient” had a significant effect in all tests, indicating that differences in BRB-N scores between MS patients and healthy controls remained significant even after controlling for the effects of apathy, fatigue, and depression (Table 5).

**Table 5:** Effect of patient, apathy, fatigue, and depression factors in the BRB-N tests

	**SRT-LTS**		**SRT-CLTR**		**SRT-D**		**SPART**		**SPART-D**	
**Explanatory variable**	**Standard estimate (β)**	***p *** **value**	**Standard estimate (β)**	***p *** **value**	**Standard estimate (β)**	***p *** **value**	**Standard estimate (β)**	***p *** **value**	**Standard estimate (β)**	***p *** **value**
Patient	−0.3135	<0.0001	−0.3465	<0.0001	−0.3189	<0.0001	−0.3591	<0.0001	−0.2706	<0.0001
Apathy	−0.1249	0.0314	−0.1154	0.0470	−0.1379	0.0191	−0.1107	n.s.	−0.1807	0.0025
Fatigue	0.1713	0.0035	0.1362	0.0199	0.1245	0.0352	0.0326	n.s.	0.060	n.s.
Depression	−0.1916	0.0028	−0.1405	0.0281	−0.1190	n.s.	−0.0803	n.s.	−0.0667	n.s.
Adjusted R-squared	0.1684		0.1668		0.1485		0.1659		0.1243	
	**SDMT**		**PASAT3**		**PASAT2**		**WLG**			
**Explanatory variable**	**Standard estimate (β)**	***p *** **value**	**Standard estimate (β)**	***p *** **value**	**Standard estimate (β)**	***p *** **value**	**Standard estimate (β)**	***p *** **value**		
Patient	−0.5251	<0.0001	−0.3823	<0.0001	−0.3858	<0.0001	−0.4319	<0.0001		
Apathy	−0.0764	n.s.	−0.0176	n.s.	−0.0108	n.s.	−0.1511	0.0058		
Fatigue	0.1340	0.0074	0.0842	n.s.	0.0673	n.s.	0.2171	<0.0001		
Depression	−0.2664	<0.0001	−0.2471	<0.0001	−0.2516	<0.0001	−0.1708	0.0046		
Adjusted R-squared	0.3933		0.2221		0.2301		0.2647			

n.s.: not significant (*p* > 0.05).

## Discussion

Some degree of cognitive impairment is found in at least half of all patients with MS, and cognitive impairment typically consists of domain-specific deficits rather than global cognitive decline [9,22]. Cognitive impairment may be affected by environmental and educational factors, and there have been no large population studies on cognitive function in Japanese patients with MS. The BRB-N is now widely accepted for use in clinical studies [23] as well as in clinical practice [7]. Furthermore, studies in several populations using the BRB-N have revealed that the battery is largely unaffected by language or cultural differences, thereby validating its use in different populations [6,7,24]. The values obtained from the healthy control group in our study were similar to those found in Dutch [6], Italian [24], and Spanish [7] populations, indicating that our Japanese version did not influence performance on the test.

PASAT is a complex task and its performance largely depends on information-processing speed and working memory, which are two important and separate cognitive processes involved in the execution of the test [25]. Although the PASAT involves a larger number of cognitive processes, the SDMT could provide a better index of information-processing speed, which seems to be more frequently impaired in patients with MS [25,26]. Further, SDMT is a good test to predict cognitive impairment in patients with MS, even in the early stages of the disease [27]. Our data demonstrate that cognitive function is impaired also in Japanese patients with MS, especially in terms of information-processing speed and attentional deficits, as shown by their SDMT and PASAT scores.

Previous studies demonstrated that physical disability evaluated by EDSS score was independently associated with cognitive impairment evaluated by the BRB-N [7,24,26]. We also demonstrated a correlation between physical disability and cognitive impairment in the present Japanese MS population. These data suggest that inhibition of relapses and improved prognosis with disease-modifying therapies will also benefit cognitive function.

Some previous studies suggested that cognitive performance does not seem to correlate significantly with disease duration [22,24]; however, longitudinal studies suggest that cognitively impaired patients experience ongoing cognitive decline [1,28]. The reason for these conflicting results remains unclear, although the proportion of patients with different MS subtypes (primary progressive, relapsing–remitting, and secondary progressive) or patient age may be important. Previous studies suggest that long-term treatment with IFNβ may protect against cognitive impairment in patients with MS [29,30]. In our study, there were not any significant differences in all 9 BRB-N tests between MS patients who received IFNβ and those who did not receive IFNβ, however, the durations of IFNβ treatment were various. It is difficult to conclude effects of IFNβ treatment on cognitive function in MS from our study, and further studies are needed about effects of DMDs on cognitive function.

In the present study, we aimed to evaluate correlations between cognitive impairment and the three factors of apathy, fatigue, and depression in MS patients. Our results demonstrate that MS patients had more apathy, more fatigue, and more depression compared with healthy controls, and decreased cognitive function was correlated with apathy and depression in Japanese patients with MS. Despite the association between cognitive variables and depression/apathy, cognitive function was impaired beyond the effect of depression and apathy. No associations between disease duration and scores on the AS, FQ, or BDI-II were found although positive correlations between EDSS and all 3 scores were found in MS patients. Other studies also demonstrated no significant longitudinal change in the Fatigue Severity Scale across a 2- to 3-year interval in patients with MS [31], and fatigue was not correlated with disease duration [32]. Together these previous and the present findings suggest that disease duration may have little association with subjective fatigue.

Apathy is one of the major neuropsychiatric symptoms in patients with MS [33]. Figved et al*.* reported that apathy was significantly associated with intrusions in patients with MS [34], although few studies have explored the relationship between cognitive impairment and apathy. We demonstrated impaired apathy in Japanese patients with MS compared to healthy controls, and a negative correlation was found between apathy and cognitive function. Future studies of cognitive function should also focus on apathy.

Fatigue is a common symptom of MS, and patients with MS often report a correlation between self-reported fatigue and their perception of poor performance on cognitive tests [35]. However, no relationship has been reported between fatigue and cognitive impairment [33,36]. Our results support these findings, and subjective fatigue may not be strongly associated with cognitive impairment in MS patients. However, differences exist between subjective and objective cognitive fatigue [37]. Furthermore, fatigue could lead to unemployment in MS patients and thus a reduction in quality of life [38], and it is therefore important to investigate cognitive function and subjective fatigue using different approaches.

The prevalence of major depression in patients with MS is relatively high [39] and this may affect cognitive function. Indeed, it was reported that depression influences cognitive performance [40], although in another study depression it was not found to correlate with cognitive function [41]. Despite previous inconsistent findings regarding the association between depression and cognitive function, our results demonstrated that depression was correlated with the individual tests of the BRB-N. BDI-II is an instrument to measure the severity of depression, not to diagnose major depressive disorder. Our data of BDI-II demonstrated MS patients scored significantly higher compared to healthy controls, and suggested that MS patients may suffer from sub-depressive conditions.

## Conclusions

Cognitive function, in particular information-processing speed, was impaired and decreased cognitive function was correlated with apathy and depression in Japanese patients with MS. However, subjective fatigue was not associated with cognitive dysfunction. Both fatigue and cognition affect quality of life for patients with MS, and we may need to consider therapeutic intervention to improve fatigue and cognition using different approaches.

## Competing interests

MN has received funding for travel and/or speaker honoraria from Biogen Idec, Bayer Schering Pharma, and Novartis Pharma, and has served on the scientific advisory boards for Biogen Idec. T. Kohriyama has received speaker honoraria from Biogen Idec, Bayer Yakuhin Ltd., and Novartis Pharma. IK has received funding for travel and/or speaker honoraria from Novartis Pharma, Biogen Idec, and Bayer Schering Pharma. YS has received honoraria for speaking from Bayer Yakuhin Ltd., and has received personal compensation for consulting services from Biogen Idec, Teijin Pharma and Novartis Pharma. HF has received funding for travel and/or speaker honoraria from Biogen Idec, Daiichi Sankyo Inc., Dainippon Sumitomo Pharma and Novartis Pharma. IN has served on the scientific advisory boards for Biogen Idec, Novartis Pharma; received funding for trips and speaks from Bayer Yakuhin Ltd., Biogen Idec, Tanabe Mitsubishi Pharma, Novartis Pharma, and received grant support from Mitsubishi Chemical Medience Corporation. S. Kusunoki has received speaker honoraria from Teijin, Nihon Pharmaceutical, Benesis, Japan Blood Products Organization, Novartis Pharma, Asahi Kasei, and Sanofi Aventis. KN has received funding for travel and/or speaker honoraria from Biogen Idec, Bayer Yakuhin Ltd., Mitsubishi Tanabe Pharma, Nihon Pharmaceutical Co., Ltd., Teijin Pharma Ltd., and Novartis Pharma. TY has served on scientific advisory boards for Biogen Idec and Chugai Pharmaceutical Co., Ltd.; has received research support from Ono Pharmaceutical Co., Ltd., Chugai Pharmaceutical Co., Ltd., Teva Pharmaceutical K.K., Mitsubishi Tanabe Pharma, and Asahi Kasei Kuraray Medical CO., Ltd; has received speaker honoraria from Novartis Pharma, Nihon Pharmaceutical Co., Ltd., Santen Pharmaceutical Co., Ltd., Abbot Japan Co., Ltd.., Eisai Co., Ltd., Biogen Idec, Dainippon Sumitomo Pharma Co., Ltd., Mitsubishi Tanabe Pharma, Bayer Holding Ltd., and Astellas Pharma Inc. JK is a consultant for Biogen Idec, and has received honoraria from Bayer Healthcare and funding for a trip from Bayer Healthcare and Biogen Idec. M. Matsui is part of a scientific advisory board for Biogen Idec, and has received speaker honoraria from Bayer Healthcare, Biogen Idec, and Tanabe Mitsubishi Pharma. YM has received speaker honoraria and research material from Novartis Pharma. S. Kikuchi has received speaker honoraria from Novartis Pharma, Boehringer Ingelheim, Kyowa Hakko Kirin, Dainippon Sumitomo Pharma, and FP Pharmaceutical Corporation, and serves on the scientific advisory board for Novartis Pharma. NM, M. Mori, TO, KM, K. Yoshida, T. Kanda, FY, SN, and K. Yokoyama declare that they have no competing interests.

## Authors’ contributions

MN was responsible for study design, data collection, and manuscript preparation. NM was responsible for statistical analysis and manuscript preparation. IK and KM were responsible for study design, data collection, and manuscript review. S. Kusunoki and S. Kikuchi were responsible for study design and manuscript review. T. Kohriyama, M. Mori, TO, YS, HF, IN, K. Yoshida, T. Kanda, KN, TY, FY, JK, SN, K. Yokoyama, M. Matsui, and YM were responsible for data collection at their respective institutions and manuscript review. All authors read and approved the final manuscript.

## Pre-publication history

The pre-publication history for this paper can be accessed here:

http://www.biomedcentral.com/1471-2377/14/3/prepub
